# ‘Recovery work’ and ‘magic’ among long-term mental health service-users

**DOI:** 10.1111/1467-954X.12020

**Published:** 2013-05-12

**Authors:** Jennifer Laws

**Affiliations:** Durham University

**Keywords:** hidden work, magical work, mental illness, psychosis, therapy, welfare reform

## Abstract

Based on an extended period of qualitative research with mental health service-users in north-east England, this article considers the various forms of ‘magical work’ and ‘recovery work’ that emerge in the lives of people living with severe mental health problems. Given the now sizeable body of literature which seeks to problematize traditional conceptual boundaries of work, the article asks to what extent these hidden and unusual work-forms might also be considered legitimate members of the category. Rather than argue for the expansion of the construct to accommodate these activities, the paper attempts simply to problematize the extent to which so-called ‘mad’ forms of work are irresolvably different to more conventional forms of occupation. In challenging notions of the psychiatric patient as inevitably inactive, new vocabularies for service-user work are explored. Concluding remarks are also directed to recent policy debates concerning ‘back-to-work’ welfare reform for long-term out of work service-users.

## Introduction: opening up the concept

This article considers how the construct of work appears in the lives of long-term users of mental health services. Through exploring three divergent narratives of work, the paper explores how even in the apparently workless spaces of mental ill health, work remains a pertinent navigational construct through which service-users order their lives. Given the now well-established trend in sociology of pushing out the conceptual boundaries of work to include various unpaid activities outside of formal employment (Glucksmann, 1995; Taylor, 2004; Pettinger *et al*., 2006), the article asks how the service-user activities described here relate to conventional work, and whether these might also comprise suitable candidates for inclusion in the category. In an important part of the argument, an exclusively medical explanation of mental distress is put to one side and the view advanced instead that what we think of as mental ill health – like work – is a fuzzy-boundaried and primarily *social* construct. To celebrate what is creative and courageous in the lifeworlds of mental health service-users as well as that which concerns suffering and disability, in what has become something of a thriving counter-tradition in mental health research, the term ‘madness’ is adopted throughout the article alongside more clinical terms such as ‘mental illness’ or specific diagnostic criteria (van Dongen, 2002; Bentall, 2003; Appignanesi, 2009).

Introducing a study of service-user work to the debate on how to conceptualize work is interesting for several reasons. As has been argued by others, the work and activity of people with mental health problems have long been ignored or pathologized (Foucault, 2001; Porter, 1987). At a discursive level, madness stands in stark opposition to the rational self-interest and productivity on which capitalist employment is seen to rest. Where the activity of mental health service-users does appear in the public eye, this is too often for random acts of violence and not often enough for the many positive contributions of mad people to society (Harper, 2005). Yet, more often still, service-users are portrayed as passive and idle recipients of what other people do to them, ignoring the possibility of ‘mad agency’ entirely (Boyle and Harris, 2009). Focusing on the work of those who appear ‘maddest of all’ – those living with and recovering from psychotic (delusional and hallucinatory) conditions – is thus at times counterintuitive given conventional renditions of what it is to be working. As I will argue later, to do so is also an ethical endeavour, joining others already working at challenging ignorance about mad work and mad workers (Parr, 2008; Grove *et al*., 2005).

However, as a second justification, attending to how service-users and the institutions of psychiatry position particular aspects of illness and recovery into vocabularies of work also provides a means of demonstrating the power and breadth of work as an organizing principle in social life – even in places which seem, as suggested above, far from the traditional workplace. With this in mind, two complementary analytic strategies are adopted with regard to the empirical material presented in the paper. In the first, which is primarily a mode of description, various forms of mad activity are recounted and comparisons made with instances of paid employment in late capitalism. It should be noted that this strategy does not necessarily seek to redefine these peripheral service-user activities within a greater category of work (as if making some ontological discovery); rather, the task is simply to disrupt the extent to which ‘mad’ and service-user forms of work are irresolvably different to more conventional occupations. In the second strategy, attention is paid more specifically to the rhetorics of work and the ways in which the linguistic choices of social actors position particular activities and identities within or outside of work-related discourse. Following the work of authors such as Jenness (1990) and Cockburn (2012) who have used similar strategies to examine other marginal work activities such as prostitution and street newspaper vending, such an approach places particular attention on the emic properties of participants' narratives. Alongside these emic and descriptive approaches, throughout the article new vocabularies of work are sought to re-describe the boundary between mad and sane forms of occupation. In the first of my three case studies, the anthropological term ‘magic’ is introduced to encapsulate what is simultaneously fantastical yet productive about certain appearances of work in delusions. Later, hybrid terms such as ‘service-user professionalism’ and ‘service-user production’ extend this vocabulary to denote how an understanding of ‘mad’ work must also encompass and exemplify more conventional notions of work and career.

In the remainder of this paper, the structure will be thus: after describing research methods and data, the article will introduce, through a series of narratives of work, three divergent and challenging forms of service-user work that emerged in the lives of people living with mental distress: the magical work that appears in delusions and obsessions; the recovery work involved in being a service-user; and the collective and semi-public forms of service-user enterprise that unfold in the contexts of niche barter economies in mental health facilities and the emerging field of service-user led healthcare delivery. Finally, a careful analysis is undertaken of the qualities of these work-like pursuits and their relation to an ever-widening construct of what it means to be working. Conclusions point to the ethics of recognizing psychic and invisible forms of work, as well as the conceptual difficulties in dissociating so-called ‘mad’ forms of work from other, supposedly saner occupations.

Some readers will note, contrary perhaps to expectations, that this article does *not* start with a concise or convenient definition of conventional work with which to compare the more liminal forms of occupation here described. Rather, following the work of Clifford Geertz (1973), the task is to provide a ‘thick description’ of the *multiple* ways in which service-users adopt concepts of work within the context of illness and recovery.

### Data and methods

The empirical illustrations used in this paper come from an extended period of qualitative research with mental health service-users in the north-east of England, conducted as part of a wider, interdisciplinary project concerning users' experiences and understandings of the therapeutic value of work.^1^ Primary research sites included three community-based mental health projects which were chosen for their divergent positions towards work-oriented recovery: a sheltered work programme housing a workshop and gardening project; a training and education centre which supported service-users in establishing independent social enterprises in the surrounding area; and a more traditional mental health daycentre which provided ‘therapeutic activities’ such as handicrafts and cookery to keep users mentally and physically active. The fieldwork spanned a 16-month period (June 2008–October 2009) during which the author visited the centres, as participant observer, friend and informal ‘helper’, once or twice weekly. Additional case studies (see, for example, the participants ‘Martin’, ‘David’ and ‘Benji’) have been drawn from semi-structured interviews conducted by the author with around fifty additional service-users and ex-users in mainstream competitive employment, recruited through a snowball sample within local service-user and self-help groups (the number of meetings with each of these participants ranged from two to six). Such interviews were recorded, where permitted, and later transcribed by the author. All participants in this article have at some point received ‘psychotic’ diagnoses: that is, conditions judged to interfere with a patient's connection to reality.

Research extracts as they appear in this article have been thematically analysed through careful rereading of field-notes and interview transcripts and through regular return visits to the field sites to ‘check out’ the validity of interpretations with participants. Narratives and quotations are drawn from a combination of verbatim interview transcripts, notes from unrecorded discussions and from close consultation with field-notes (all indented or ‘speech-marked’ phrases are verbatim quotations). An important aspect of the research was the researcher's own status as someone who lives with a mental health diagnosis and who has used acute mental health services – a fact of which participants were aware – and as such, the research shared many qualities with participatory and service-user led methodologies. It should be noted, however, that extracts have been selected to demonstrate the *range* and *breadth* of experiences of ‘mad work’ – as well as points of conceptual intrigue within the topic – rather than to convey what are necessarily dominant viewpoints or experiences among the mental health community.

The three forms or manifestations of service-user work presented below are just one of many possible interpretations of the data and, following the work of van Dongen (2002), are best understood as sympathetic and creative explorations into the alternative worlds of psychosis, rather than robust or representative typologies. As per other outputs from this research project (Laws, 2009, 2012), in lacing empirical and theoretical debate together, the paper continues in a commitment to philosophically informed discussion about work and mental distress which ‘is supported throughout its texture by cross-reference to experience’ (Collingwood, 1933: 51).

## Three narratives of work: the appearance of work in the lifeworlds of psychosis

### Magical work

I am a pilot, I am going to join the Forces, I am a pilot, I am a pilot, yes! A war, it is a secret, a secret test pilot. You won't see the engine because I am travelling too fast. (Sam, participant in the sheltered work project)

A first and most vivid manifestation of the kinds of ‘mad’ work that I want to discuss in this paper is the appearance of unusual and imaginary work experiences in the alternative worlds of psychosis and obsession. Whilst ‘psychotics’ (ie those who are deemed to have lost touch with reality) are generally considered the hardest to help in conventional occupational therapy programmes, conversely, the so-called ‘clinical’ presentations of psychosis – the manifest content of delusional beliefs and obsessions – are often about work. According to epidemiological research, amongst the commonest themes in delusions are ‘being on a mission’; ‘following a calling’; ‘having especial abilities’; ‘accomplishing extraordinary achievements’; and ‘being a self-made man’ (Leff *et al*., 1976; Junginger *et al*., 1992; Kim *et al*., 2001; Bentall, 2003). Following the work of the anthropologist van Dongen (and, before her, Lévi-Strauss), such psychical vocations can be thought of as a kind of ‘magical’ work: undertakings that are formally imaginary yet which, through the mimetic rearrangement of culturally available myths and symbols, have power to bring things into being in the real world (a form of conjury since such transformations of selves and identities appear to emerge from nothing).

Some of the participants involved in my research have believed themselves to have some magical work to do. To take some examples: Sam, a participant at the sheltered work scheme on ‘day release’ from a local in-patient hospital, is flying a secret new fighter-jet in the Royal Air Force. Her duty is to protect the nation. The mission is undercover, but we in the workshop are allowed to hear about it. In my first encounter with Sam at the day programme, she bursts into an in-progress focus group on mental health and employment with participants whom the staff had selected for me to speak with. ‘I have a very important job’, she says and pulls up a chair to join the group. Martin, a successful and well-liked businessman, must hunt for nylon shirts. Following a period of acute illness when he became worried he might be supernatural, wearing the manmade fibres now assures him he is a ‘real’ human and the shirts form an essential part of his daily attire. Given their increasing scarcity, the hunt for new shirts takes him on a national tour of charity shops and has also led to the start-up of an internet-based retro fashions outlet which he manages in his spare time. The work is enjoyable but not without its anxieties, both because of the time it costs him from his business, and the fear that supply might run dry. Vera, one of the daycentre's most loyal members, resists any active part in the ‘therapeutic activities’ programme, except to organize the lottery syndicate. The day is punctuated by her shuffles around the tables, asking ‘Got your 50p?’ long after all likely participants have contributed or bowed out (and sometimes when there is no lottery draw to enter). People rarely attempt to dissuade Vera or disturb her in her work: to ask Vera for an interview, or to invite her for lunch or play bingo, results in only an angry shaking of the tin – ‘I got to do the syndicate, ain't I?’ David, a young man re-entering employment after several months of hospitalization, must ‘check’ (lightly tap with his knuckles) the walls of his surroundings at frequencies instructed by internal voices. The work has sinister responsibilities: completing the rituals prevents ‘bad’ things from happening (the apocalypse included); as an apparent residue from his previous job at the bus depot, the routine checks are also essential for the punctual operation of the public transport system.

For the purposes of this paper, how should we interpret these ‘magical’ forms of work? In classic psychoanalytic understandings of delusions, the fantastical forms of work in psychosis and phantasy are little more than childlike rejections of an adult work ethic: regressions to an early and more preferable state of infancy in which the developing self experiences an ‘omnipotence’ of thought in which wishes are automatically gratified – as they almost are for the newborn (Weil, 1959). The florid contents of delusions, with their frequent renditions of heroism and triumph are thus but elaborate ego defences to protect the psychotic individual from the more painstaking work of the ‘real’ workaday world (and such views were certainly expedient in maintaining the mid-20th century image of the psychiatric patient as indolent and petulant).

However, more contemporary evidence from cognitive neuroscience and social epidemiology gives reason to be cautious of such hurried dismissals of the work of delusions and obsessions. First, an increasing body of psychopathological research suggests that, clinically speaking, delusional beliefs and sensory hallucinations share far more in common with ordinary cognitive processes than formerly imagined (Roberts, 1991; Freeman and Garety, 2004; Bell *et al*., 2006). Drawing on data which examines the cognitive and emotional differences between psychotic and non-psychotic belief systems, such studies suggest that the basis of delusional thinking may be less a psycho-emotional *withdrawal* from reality, and more a faulty attempt at *engaging with* reality. In a second band of research, cross-cultural and historical studies suggest that the type of work prevalent in psychosis is shaped by the kind of occupation respected by society at that time – an unexpected finding if we believe that the mad are in some profound way *anti*-work. In Burnham's historical analysis of case records in a mental hospital in Tasmania (1830–1940), for example, patients built railroads and farmed potatoes in their delusions – forms of labour much-needed in the industrializing colony (Burnham, 1980). In another study, Kim *et al*. (2001) demonstrate how the presentation of schizophrenia in contemporary Taipei, Seoul and Shanghai varies with broader cultural anxieties surrounding capital and livelihood. In Shanghai, delusions on the themes of business were most frequently associated with grandiose beliefs such as being a millionaire – apparently consistent with a broader Chinese obsession with money in the recently opened market. In Taiwan and South Korea, however, delusions about being robbed, cheated or swindled in business were more common, reflecting national anxieties about the instability of free capitalism among the Asian Tigers. In my own research, the stories of Sam and the others support this thesis. In a society where the armed forces are celebrated as ‘heroes’ amidst unseen enemies, the work of an imaginary fighter pilot has a good level of cultural legibility. Similarly, in an economic environment in which inefficiency can indeed have ‘apocalyptic’ effects for businesses and their employees, David's anxieties about performance and punctuality also resonate with wider socioeconomic concerns.

Observations about the ‘cultural coherence’ of delusions offer little guidance on whether the contents of delusions should be considered an authentic form of occupation. However, with the above evidence in mind, if delusions are full of work, it seems more sensible to suggest this is because the mad understand that work is important, rather than because in some way they are rejecting or transgressing it. Magical work is not always successful work and Sam and the others do not always reap the rewards for their psychic endeavours in the imaginary realm (a point which van Dongen makes in her own ethnography from the locked wards of a Dutch psychiatric hospital). Yet magic transforms people from ‘patients’ into ‘pilots’ and provides the means for even the most occupationally deprived to seek job satisfaction. For van Dongen and myself, ‘magic’ thus offers some justice to the work that appears in delusions, by showing what is creative and productive in psychosis, as well as is what is incalcitrant and perverse. Through stressing a *surplus* rather than absence of meaning in the work of psychotic people (the cultural legibility thesis), ‘magic’ articulates what is public about private madness. It also shows how mad work exercises power over an ordinary workaday world yet, unlike some more conventional forms of paid employment, simultaneously resists being reduced to its laws and regulations.

### Recovery work

I was just trying to put myself back together, really. That's what my energies were going into. And what was happening here [in the sheltered work scheme] was helping that, but getting better was a work and an end in itself (Kev, participant in the sheltered work programme).

If work reveals itself *in* madness, the combined effort of living with and recovering from mental distress can bring its own form of work also.

Therapy has long been understood as a kind of psychic recovery work (Riesman, 1950; Bartlett, 1973). For Freud, the process of ‘working through’ (or, the therapeutic process of repetition) was a central mechanism through which psychotherapy sought to replace maladaptive unconscious drives with conscious understanding (Freud, 1914). According to the theory, clients' difficulties had to be faced in a number of different contexts both with and without the therapist's support before change could be said to be authentic. In ‘working through’, a double work emerged: patients both worked *through* the repetitions (up to a point at which learning was complete), yet also *worked* through the repetitions; that is, the repetitions and their constant reinterpretation were *acts of work themselves*. In more action-oriented behavioural therapies such as cognitive behavioural therapy (the current ‘gold standard’ in the treatment of depression), recovery is also described as an act of work. Indeed, the extensive use of individual *homework* tasks such as mindfulness training or ‘thought logging’ (a widely used technique of recording and cataloguing moments of maladaptive thinking) is considered by many to be the driving mechanism of CBT as a therapeutic intervention (Addis and Jacobson, 2000).^2^ However, it would be a mistake to suggest that these highly prescriptive forms of work taking place within professional therapy are the only work undertaken in recovery. As Kev describes above, ‘getting your head round things’ is a significant form of work in itself, wherever it is done; and whilst this might not always generate effects in the outside world, it nonetheless has the capacity to be time-consuming and challenging. Finally, as others have argued, becoming ill and negotiating a new status of ‘psychiatric patient’ also generates all kinds of additional cultural ‘face-work’ and ‘identity work’ in adjusting to a new social self (Goffman, 1961; Aneshensel, 1999). Goffman's ‘moral career’ of the mental patient primarily used the notion of career to describe the predictability of the social stages through which a psychiatric patient passed in the course of his illness. Yet, as more recent users of the concept have shown, such careers are similarly work-like and necessitate frequent psychological and cultural upheavals.

If the above examples all describe predominantly intra-psychic labours, being a psychiatric patient and engaging with the myriad requisite services brings with it more down-to-earth forms of work too. Using Star and Strauss's concept of ‘background work’ (a type of invisible work in which the workers themselves appear visible, yet the work they perform goes unnoticed), Unruh and Pratt (2008) provide a revealing account of the multiple forms of behind-the-scenes work undertaken by patients in an outpatient cancer unit, such as travelling between care sites, finding information, managing medication side-effects and communicating with professionals. In the in-between workplaces of my own research, similar activities took place – and in the instance of the social enterprises and sheltered workshops such background patient activities had to be completed before and around the ‘real’ work of gardening, catering and packing.

Following the philosopher Peter Winch (2007 [1951]), perhaps the extent to which the numerous activities undertaken by patients really constitute a form of *work* is revealed best through an examination of the language in which they are discussed. Just as psychotherapy adopts an explicitly work-related vocabulary to describe its processes, in the daycentres service-users sign *care contracts*, attend *case conferences* and *annual reviews* and, for older members approaching transfer to specialist geriatric facilities, often speak of *retiring* (indeed, the generic shift in service provision from the term ‘patient’ to ‘service-user’ or ‘client’ is redolent of this heavily work-infused discourse). A recurring theme in this work-driven vocabulary is the reference to users *managing* themselves. Discourses of patient self-management are undoubtedly modish in current UK health policy (see, for example, the continued expansion of the publicly funded ‘Condition Management Programmes’ for patients with long-term health complaints), but it is notable that, again, service-users also speak explicitly and extensively of the managerial duties undertaken through living with mental distress. This is encapsulated most clearly in an interview extract with Martin, the businessman who we met in the previous section:

*Martin*: I can sometimes actually feel the electrodes of my mind fizzing away, and that's when I might decide to pop an extra haloperidol [anti-psychotic drug] or just chill out for a bit or go for a walk, but generally just lying down horizontal is the best way – so it's really about managing it.

*Myself*: Hmm. I've noticed you've said that word ‘managing’ several times today?

*Martin*: Yes I *have* said it several times because I manage the illness in the same way that I manage other things like I manage staff and manage finance and manage cash-flow or whatever it might be. It's just another aspect of my life that I need to take control of, because when you do lose control, that's when you end up in hospital.

In the instance of patient self-management, the *material* vocabularies of recovery are similarly work-related: one participant, under the advice of his occupational therapist, used a ‘Blackberry’ (smartphone and digital organizer) to coordinate his busy schedule of medical appointments, medications and rehabilitation activities. Similarly, at the training centre, a six-week course in ‘self-care and healthy living’ gained accreditation with a local further education college during the period of my research. In the training centre, another important nexus of recovery work was found in the computer room. On the computers, users worked through CBT software packages, researched their psychiatric conditions or accessed peer-support on the Internet, or else updated computerized forms of the thought-logging technique described above using specifically designed interactive spreadsheets. Again, this ‘mad’ work interconnects frequently with everyday work and concerns about employability, with more than one service-user commenting that the computer skills gained from learning to use the thought-logging software had been more useful than the more formal IT training provided by an external ‘skills for work’ tutor.

As a final comment on patient self-work, it should be noted by the reader that the examples offered in this subsection stop strictly at the *descriptive* level. Just as conventional paid work can be both psychologically rewarding and damaging to workers, to observe that self-care and recovery is work-like provides little clue to its human consequences. For Freud (a romantic in this respect), the kind of work imagined in ‘working through’ was itself a therapeutic work: not the drudgery or danger of the factory, but a noble and uncomplaining lifework (Kirschner, 1996). Such similarly romantic notions of therapeutic work were noted by some of my participants too: in the gardening project, for example, Lou explained how the past few weeks of digging out thorny bushes and replacing them with softer flowering plants seemed an appropriate metaphor for the mental cultivation that had been taking place in his head. Yet in more contemporary work metaphors such as patient self-management, the kind of work imagined is harder to unearth. For Martin (a manager in real life) thinking of his self-monitoring strategies as managerial duties provides a greater sense of control and agency over his unusual mental experiences. However, just as it is in the real workplace, a fine line exists between the apparently therapeutic self-management described by Martin and the more pervasive micro-management of workers seen by many critical scholars as characteristic of a neoliberal governmentality. Recognizing what is work-like about being a mental health service-user is thus an effective response to the assertion that the ‘mad’ are ‘not working’. However, extending this re-description to an analytic discussion on the *value* of this ‘work’ is more limited in application: just as other forms of work are varied and contested, the slipperiness of the concept in the outside world also becomes reproduced in the mental spaces of madness.

### Service-user production

You know, I've not got a job but I always seem to be on my feet, because I am on the service-user committee now and I'm doing like the patient coffee mornings – once a month I've got them at my house. And we got a rota for visiting those who are bad [local colloquialism for ‘unwell'] so I'm doing that because the CPNs [community psychiatric nurses] don't get out to you much, so I do shopping and visiting for folk who are bad. (Sheila, housewife and service-user)

As a final (and briefer) discussion, a third presentation of work which is important to highlight is that of service-user markets, or to put it differently, the potential for ‘mad’ work to transcend the level of the individual and form economies of trade and service-provision within the niche subcultures of psychiatric survivorship. In contrast to the above examples, in this instance it is less the functions of work (selling, providing, delivering) which are divorced from supposedly saner forms of work, than the self-sufficiency of ‘mad’ markets within the mental health community that is worthy of note.

A first, provocative example of this is a consideration of the various ‘black’ or underground economies present to varying extents at each of the daycentres I visited – engaged in by a minority, but known about by most. In these semi-institutionalized spaces, exaggerated by the limited freedoms of some participants to leave the grounds unattended, complex networks of trade were described by participants for the introduction and circulation of specifically niche commodities within the projects (and then frequently back into hospital wards). Such commodities, condemned by conventional psychiatry but an essential part of day-to-day life for some service-users, included laxatives for bulimic patients and razor blades for self-harmers, as well as benzodiazepine sedatives (a frequent ‘drug of choice’ for psychiatric patients following dependencies developed during legitimate use on the wards), and could be sold for cash or exchanged as part of a barter economy. Potentials for the sourcing of more idiosyncratic needs also existed: patients at the daycentre often discussed ‘Betty’, an obsessive collector of hats and postcards, who spent most of her time in high security care. Whilst Betty's family had long given up on her insatiable need for new objects, a ready crew of ex-ward mates appeared endlessly able to deliver the goods at visiting time in return for cigarettes (and, of course, the cost of the item repaid): as above, a work-based vocabulary prevailed; ‘I'm on a job for Betty’ was common shorthand for such excursions.

Yet, if bringing to the foreground these underground enterprises further pathologizes service-user work, a more publicly acceptable case might be the increasing market-based outsourcing and delegation of support and rehabilitation work from state-led mental health and social services to third sector service-user organizations, through which patient networks begin to behave not only as channels of peer support, but as professional bodies able to receive care loads from public sector contracts (Boyle and Harris, 2009). Heralded by policy-makers as a significant lever in the future of public services reform (and already observable in some of the organizations involved in this study), such ‘partnerships’ between conventional state-funded care providers and patient networks utilize not only the enthusiasm of service-user communities but also their expertise and efficiency at undertaking challenging projects: speaking to a service-user coordinator of such projects, for example, I was told how the network had been approached by statutory services to facilitate a self-help group for people with personality disorders (conventionally considered among the hardest to engage) due to the network's strong record for work with ‘difficult’ clients.^3^ Such spaces of ‘service-user production’, as they have become known, open the way for not only new forms of ‘expert patients’ but also new forms of service-user professionalism and, with them, new spaces of patient work. Unlike the forms of magical and self-work described in earlier sections of the article, these public forms of service-user contribution demonstrate the ability of service-user work to transcend the intra-psychic realm, and in the argument which will take over the final section of this paper, ask the question, to what extent are ‘mad’ forms of work truly separate from other forms of supposedly sane occupation?

## Discussion: classifying service-user work

As discussed in the introduction of this article, problematizing the conceptual boundaries of work has been a recurring concern in the sociology of work and beyond, most often in the form of opening up the category to non-traditional kinds of ‘worker’ previously excluded from its reaches. Prominent examples include feminist treatments of homemaking and caring (Oakley, 1974); examinations of unpaid work in the voluntary sector or black economy (Taylor, 2004); and, further afield, anthropological studies of non-capitalist societies, exploring the work-like qualities of subsistence modes of production (eg Kaplan, 2000). Joining such literatures, a host of additional papers have emerged on cultural ‘work’, consumption ‘work’ and other activities traditionally considered outside of the construct. An overarching strength of such literatures is their collective power to reveal how traditional notions of work ‘marginalize and devalue’ the activities of atypical workers (Taylor, 2004: 30) and to signal critical arenas for future work scholarship. However, despite such potentials, such ‘new sociology of work’ contributions retain an uncertain position in the wider discipline: on the one hand, experiencing only limited uptake in more mainstream work-related studies (which instead remain to a large extent restricted to the study of paid employment); on the other, raising questions – even among sympathizers – about the *usefulness* of such radically extended and destabilized concept of work (see Watson's comments, cited below, for an example). With such comments in mind, how should we best interpret the ‘mad’ forms of work described in this article, which seem so simultaneously workful and workless?

### Considering the criteria

If an essential criterion of work is to bind the individual to a material reality – and this was indeed the basis of Freud's therapeutic conception of work – then on first glance the magical work of delusions and obsessions (the first of my three categories) cannot be real: David's ‘checks’ in the hospital will ensure the timely arrival of buses no more than Sam's aeroplanes will ‘really’ protect the nation. However, whilst this might appear a convincing argument, a precursory survey of the contemporary job market demonstrates that, conversely, much conventional paid work also fails to match this ‘reality’ criterion. The rise of the information and service sectors – those which are often definable by their very retreat from material production (particularly of the kind that was offered in the workshop or factory) – has led some to class the increasing *im*materiality of labour as a key harbinger of ‘economic postmodernization’ (Hardt and Negri, 2000: 280). Nor does the quality of fantasy which characterizes much delusional work necessarily set mad work apart from sane. Whilst classic critiques of capitalism have long stressed the mythical elements of business and the market (eg Arnold, 1937), striking contemporary examples such as the ‘virtual sweatshops’ of computer gamers in the developing world who are hired to complete the easy levels of online games on behalf of money-rich, time-poor Westerners highlight the extent to which capitalist waged labour can also operate on a primarily *fantastical* level (*The Observer*, 2005). The concept of ‘magic’, introduced to explain the mad work of psychoses, has similar utility in describing the ritual modes of visioning and ordering the world that are common to late global capital. Like the work of the service-users in my study, such sorcery does not always have happy consequences: the financial models of the bankers in the lead up to the 2008 financial crisis, which failed so dramatically to relate to the ‘real world’, capture a vivid moment at which this magical ordering fails.

As a further objection, whilst it is tempting to assume that delusional work (ie the work of psychotic patients) is inevitably intra-psychic and otherworldly, from the more grounded examples of this ethnography it can be demonstrated that, like non-mad work, mad work demonstrates a host of relations with the outside world. Whilst Sam's aeroplanes do not exist in any real-world sense, by contrast, although Martin's beliefs about the powers of nylon might make little sense to us, materially (no pun intended), the shirts are undoubtedly real. Indeed, for Martin, the crucial factor of the shirts is the assurance they provide of a tangible, manmade reality. The online fashion store demonstrates a high level of activity, ingenuity and skill, as well as generating economic profit. Furthermore, whilst the obsession with nylon and the pilgrimages to Newcastle's charity shops might be considered a *private* work and a *private* madness, an actor network theory perspective, for example, might see instead the interface of the Internet business as laced with transactions between a private and public sphere, conjoining psychic, virtual and material economies for Martin and his customers. In many ways, then, it can be demonstrated that the magical work that appears in delusions is more on a continuum with ordinary employment than previously imagined.

To turn to the second of my ethnographic examples, the ‘self-work’ of being a service-user presents a different set of challenges to the murky task of defining work. In a now famous analysis on the subject, Hakim (1996) draws on a long heritage of work theorists to arrive at what she calls the ‘substitution rule’ or ‘third-person criterion’ to distinguish between activities which constitute work and those which do not. According to the rule, an activity is deemed to be work only if it could be undertaken by a substitute (ie someone other than the one benefiting from the activity) without the task losing its value; conversely, if an activity would lose value or fail to ‘make sense’ if a substitute took over, then in Hakim's framework, this is not work.

Following this line of thought, a ‘working on the self’ seems highly problematic to ‘real’ work as defined by Hakim. In the kinds of work that service-users do to and for themselves, not only do the kinds of task described (regulating one's emotions, modifying one's thoughts) have an intimacy that makes them intuitively inalienable from the self, since worker and object of the work are one and the same, the premise of ‘third person substitution’ appears practically as well as philosophically impossible. Yet, in the instance of mental health service-users, this can be problematized again. Given that, ordinarily speaking, a professional therapist is considered to be doing legitimate work, how are we to distinguish work on *someone else's* self as necessarily different to work on one's *own* self? Remote technologies such as the CBT computer programmes described above further break down this distinction since, whereas in traditional face-to-face psychotherapy it might be argued that the client is *receiving* a service (in the same way that she might receive a massage or pedicure), in this instance, whilst the work may be managed by the computer programmers and therapists, the in-the-moment labour of therapy seems undertaken by the patient solely. Similarly, if the original feminist premise is valid that informal *care*-work (eg in the home) is equal in value to paid employment in the care sector, then why should the particular forms of self-care that a person with mental health difficulties might do to or for herself *a priori* be excluded because the carer and recipient of the care are one and the same? As an unusual example to illustrate this latter point, one of my interviewees reported an occasion where during a ‘manic’ phase, she saw advertised a free ‘training and treats’ day for carers of the long-term mentally ill. The woman applied, listing in detail the myriad tasks she did for herself to manage her difficulties. The application went undetected, but on disclosing her particulars to other participants on the course she was asked to withdraw, since the (literal) self-centredness of her work had offended other presumably more legitimate carers.

In an age where the boundary between personal development and formal work has become very blurred, the philosophical concept of psychological self-work becomes even more relevant. In a conventional framework, a client in counselling or group therapy would not be considered to be working – yet, an equivalent individual taking part in a highly similar form of self-reflection and psychosocial learning, as part of (for example) personal or professional development within the workplace or a ‘kaizen’ continuous-improvement ring would likely consider such activities to be part of their working day. Whilst a convincing counter-argument might be that, in this example, the person in group therapy is developing herself for her own benefit whilst the employee is developing herself for the benefit of her employer, two points cast doubt on this position. First, if personal development in the workplace is *only* for the profit of the employer, then to what purpose is it often *advertised* in recruitment materials as an employee *benefit* (the covering letter for my recent teaching qualification describes the certification as a ‘valuable portable asset'!)? Second, whilst people enter therapy for many different reasons, it is also acknowledged that on multiple levels therapy *does* seek to return people to their regular occupations: famously, Freud said that the key aims of psychoanalysis were to enable the patient to love and to work (Smelser and Erikson, 1980). More contemporarily, both publicly and privately funded schemes such as Increasing Access to Psychological Therapies (IAPT) and Employee Assistance Programmes (EAPs) for work-based counselling explicitly seek to reduce unemployment and absenteeism in the British workforce (as Jed, a healthcare worker and EAP recipient of work-based counselling remarks: ‘I do the sessions in work's time not mine because that's what they [the employers] say: it's in their best interests that I'm keeping on top of things and I'm not off on the sick so the therapy's part of my job, kind of’). In this sense, following the logic of Glucksmann and others, in repairing and replenishing capitalism's labour supply, therapy (like Kaizen, mentoring or PPD) is also a form of self-development that simultaneously supports economic ends.^4^

Finally, the work underpinning ‘mad’ markets and service-user production seems most of all the three narratives of work I have discussed here to be ‘proper’ work, conventionally described. Such work acts upon a real world, addresses clearly articulated social needs, and shares much in common with more conventional forms of work. Yet whilst *recovered* ‘mad’ people working in a related field (eg those who sacrifice their membership to the mental health service-user community and retrain as mental health professionals) readily claim working status for their contributions, the contributions of current psychiatric patients to service delivery through avenues of consultation and peer-support struggle to gain similar recognition. As Benji, the service-user coordinator in the above section, describes (after outlying the considerable responsibilities of her career): ‘Still my family say to me, “when are you going to get a proper job?” I don't think they see me as “gainfully employed”.’ It is my argument that this persistent under-recognition of ‘mad’ work – even in the case of the productive and professional spaces of co-production – is not only a case of a generic undervaluing of unpaid work in contemporary society (Glucksmann, 1995; Levitas, 2005), but also evidence of a more profound difficulty in aligning the apparent unpredictability and intimacy of madness with the efficiency and manageability of the productive capitalist workforce.

## Conclusion: navigating work narratives

In this paper, I hope to have offered three intersecting contributions to the sociology of work and allied disciplines. In providing a ‘thick’ description of the ways in which work appears (consciously and unconsciously) in the narratives of mental health service-users, the paper shows how even in the traditionally workless spaces of psychiatric recovery and mental health day-care, work remains a relevant and important construct through which service-users understand and navigate their lives. Second (and relatedly), the paper shows how in a culture of ‘back-to-work’ for welfare recipients, the construct of work becomes a strategic device through which service-users legitimize a series of economic and non-economic activities traditionally outside the work remit: whilst Sam's expressions of her pilot career might best be seen as a *longing* for work, conversely, in the second and third of my three work narratives, service-users speak of work *assertively*: in classifying the activities of recovery and co-support as work, they say ‘my activities are important and effortful social contributions’ – evidence again of the normative power of the work ethic in society. Finally, by exploring the commonalities between ‘mad’ work and more conventional forms of work, the paper adds to the growing literature which stresses how madness and sanity exist together on a single spectrum (Bentall, 2003).

There are reasons to be cautious about promoting the various forms of service-user activity described here as work. Simplistic tales about mad people being better workers than others need to be avoided, as do crude etiological accounts which posit mental illnesses as straightforward outcomes of a pathologically over-active capitalist work ethic. Moreover, as many participants in my ethnography would be keen to stress, this paper strictly does not uphold the suggestions that mad work, if it is work at all, is an appropriate or fulfilling alternative to quality paid employment: as has been stressed in repeated large-scale empirical studies, many long-term out-of-work service-users are highly motivated to join the labour market and despair of the multiple barriers they face (Grove *et al*., 2005). Yet, perhaps most significantly, the danger of demonstrating what is work-like within madness is that it reproduces the idea that only that which can be shown to be work has value. Watson remarks of the potential in highly destabilized understandings of work for ‘wildly imperialistic ambitions’ in encompassing ever more areas of human activity (Watson, 2009: 870). In the intimate spaces of the psyche described here, perhaps such imperialism is most objectionable: certainly, to the extent that capitalism is critiqued as an unbridled force of governance over the human soul, the creativity and recalcitrance of ‘madness’ (at least on a cultural level) offers one of the few remaining safe-havens from a relentless capitalist work ethic.

However, notwithstanding the above reservations, there is nevertheless an ‘ethics of description’ at play in bringing to focus the tremendous work of madness and recovery. In an age where (like it or not) work is posited as a moral duty, an acknowledgement of mad work offers a more respectful position towards mental health service-users and draws attention to the busyness and competence of even the most apparently ‘workless’ members of the mental health community; where such work is tinged more explicitly by sadness (Sam's imaginary aeroplanes, for example), such narratives have similar power in revealing the importance of work for occupying more readily accepted identities and statuses. Yet such (re)-descriptions must be always ironic (as is much of ‘madness’ itself), in that they stress what is workful about work and yet simultaneously concede that work itself is a fractured term that is frequently ‘not working’. The complex relationships between work and mental health are important contemporary research topics in the social and clinical sciences with direct impacts on policy and practice. A greater attention to ironic forms of mad work might contribute to understandings of why conventional ‘back-to-work’ policies often do not work and, conversely, help ring-fence a safe space for people like Sam and Vera, whose recalcitrant work in the alternative worlds of psychosis prevents concurrent engagement in a more ordinary workaday world.

## Notes

1 Approval for the fieldwork was granted by the research ethics committee of the author's institution, under the guidance of the relevant National Health Service Research Ethics Committee. All names are pseudonyms.

2 Rather than existing as stand-alone units of work, such therapeutic tasks also interact with the delusional forms of work described above. David, for example, describes how the magical work of preventing the apocalypse is interjected by the therapeutic commitment of chronicling his compulsions in one of the above mentioned ‘thought diaries’. In his words: ‘Well, it takes a lot out of your day, like. You do your thing [ie act out your compulsion], so I check the walls and everything. And then I have to go and write it down in this diary what the [occupational therapist] has given me to record that I've had an episode [laughter]. By the time I've done that and written it all down; and you have to put what you thought, what you did, and my writing's not that good anyway – well, after that, sometimes it's time to do another check! [More laughter]’.

3 These formal work contracts take place alongside more bottoms-up and grassroots projects which emerge spontaneously to meet local needs; see Laws (2009) for a fuller description.

4 A more cynical rendition of this story is the classic ‘anti-psychiatry’ claim that psychiatry and psychotherapy act as agents of the state to fashion souls fit for capitalist labour; yet it is not necessary to support such views to follow this argument.
